# The Notched Stick, an ancient vibrot example

**DOI:** 10.1371/journal.pone.0218666

**Published:** 2019-06-26

**Authors:** Marica Broseghini, Clara Ceccolini, Claudio Della Volpe, Stefano Siboni

**Affiliations:** Department of Civil, Environmental and Mechanical Engineering (DICAM), University of Trento, Trento, Italy; University of Birmingham, UNITED KINGDOM

## Abstract

An intriguing simple toy, commonly known as the Notched Stick, is discussed as an example of a “vibrot”, a device designed and built to yield conversion of mechanical vibrations into a rotational motion. The toy, that can be briefly described as a propeller fixed on a stick by means of a nail and free to rotate around it, is investigated from both an experimental and a numerical point of view, under various conditions and settings, to investigate the basic working principles of the device.

The conversion efficiency from vibration to rotational motion turns out to be very small, or even not detectable at all, whenever the propeller is tightly connected to the stick nail and perfectly axisymmetrical with respect to the nail axis; the small effects possibly observed can be ascribed to friction forces. In contrast, the device succeeds in converting vibrations into rotations when the propeller center of mass is not aligned with the nail axis, a condition occurring when either the nail-propeller coupling is not tight or the propeller is not completely axisymmetrical relative to the nail axis. The propeller rotation may be induced by a process of parametric resonance for purely vertical oscillations of the nail, by ordinary resonance if the nail only oscillates horizontally or, finally, by a combination of both processes when nail oscillations take place in an intermediate direction. Parametric resonance explains the onset of rotations also when the weight of the propeller is negligible. In contrast with what is commonly claimed in the literature, the possible elliptical motion of the nail, due to a composition of two harmonic motions of the same frequency imposed along orthogonal directions, seems unnecessary to determine the propeller rotation.

## Introduction

Screw loosening is a common practical experience; less common may be a spectacular case as the rotation of an ancient Egyptian statue in the Manchester museum [1] in its glass container without any apparent external action. In both cases it is possible to guess that the environmental vibrations contribute to the apparently autonomous movement of the object along a circular pathway centered on its rotation axis.

In the scientific literature there are at least two topics related to this same basic physical phenomenon. The first one is the “vibrot”, a term recently introduced in [2] indicating a designed method and a corresponding device to carry out such a mechanical transformation from undesired environmental vibrations to a rotation movement[3–10] (e.g., to insert and tighten a screw).

**Fig 1:**
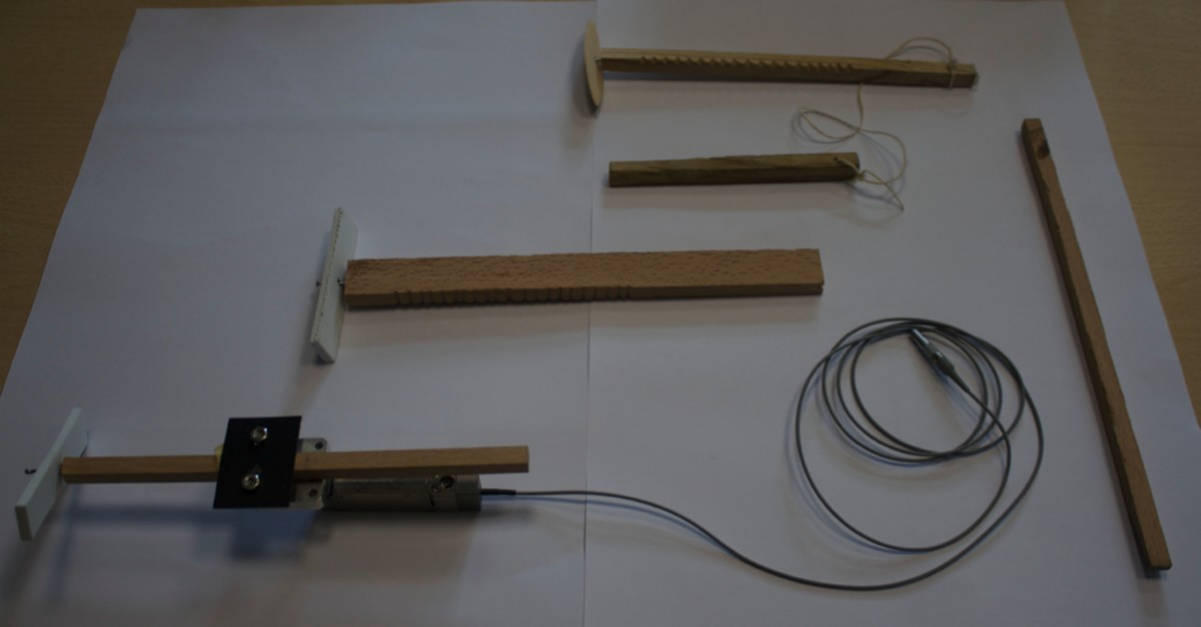
Some examples of Notched Stick. The typical appearance of the Notched Stick (NS): (from the top) a commercial one completely wood made; a NS with a rectangular stick and a plastic rotor without a microbearing; a NS mounted on a piezo, consisting of a square stick and a plastic rotor endowed with a microbearing.

The second one is an old mechanical toy, known with various names: Notched Stick, Hui game, Girigiri-Garigari, Gee-haw whammy diddle, Bozo-bozo, whose typical appearance is shown in Fig 1. This one will be the object of the present paper.

Common mechanical toys may have a great education value, often allowing to easily explain both simple and complex physical concepts involved in their working principle. For this reason, they are not only intriguing for children but also for scientists.

The Notched Stick generally consists of a notched stick made of wood on which a propeller loosely fitting a nail and free to spin, is fixed; upon moving a dowel back and forth across the notches, the propeller rotates (see Fig 1).

Of course, some details may be different: the section of the notched stick may be squared, rectangular or circular, the materials used for it may be wood, plastic or metal, the notches may be produced along one side or on the edge. These features do not change the basic working mechanism but however may affect many details of the device behaviour. A short summary of the main literature is given in the following.

To our knowledge the first scientific paper devoted to this toy has been published in 1937[11]. The author, R.W. Leonard, using a rectangular section notched stick, proposed both a mechanism for the rotation, depending on the combination of two perpendicular linear harmonic vibrations, and a mechanism to control the rotation sense, based on the edge used for the stroking action.

20 years later J. S. Miller, in 1955[12], observed in a short note that the stroking modality controls both the speed and the direction of the propeller rotation, which stops when an out of phase vibration is produced. Thus, he concluded that: "*the rotation is clearly a matter of resonance and forced vibrations*"[12].

In the same year, in another short note E.R. Laird [13] described a patented “Indian mistery stick” “with a square cross section and notches across one of the edges”; he also proposed to use the finger position on one or the other edge to change the rotation direction. Laird writes that “*the action in this case is not due to the resonance in the ordinary sense of the term*”.

In the following year G. D. Scott [14] in a more detailed analysis uses a stick with a circular section; he accepts the fact that it is possible to control the direction of rotation but he poses again the two basic questions: why does the rotor turn at all and what is the mechanism for controlling the direction? In his opinion “*the direct cause of the rotation of the rotor is most certainly a circular or elliptical motion of the nail which serves as its axle*”; he excludes the role of an off-center or elliptical hole in the rotor, as well as that of a resonance effect.

He claims that the circular motion is caused by the oscillation of the nail determined by the notched stick and distorted by the action of the thumb of hand or by fingers in a circular or elliptical motion. This same mechanism allows the direction control.

20 years later S. S. Welch [15] relaunched the question. Welch writes that a square section stick will not rotate if a finger is not used to act a pressure on one side of stick, while rectangular stick will.

In a further paper published 10 years later G. J. Aubrecht II [16] repeats Leonard’s claim that the Notched Stick is a by-product of native American culture. Moreover, he indicates a wrong reference but for an interesting idea: “*Scott suggests that the propeller is driven in the same way keeps a hula-hoop turning*”. In fact such a suggestion is not present in Scott’s short note, but we believe this is a very interesting hint, reconsidering the importance of the shape of the propeller hole where the nail is placed through. Aubrecht assumes that the spin of the propeller originates from the presence of an elliptical motion of the nail, imparting a torque to the propeller every time the two bodies are in contact. In 1988 H. J. Schilchting and U. Backhaus [17] also have provided an analytical description of the phenomenon proposed by Scott.

In 1992 a paper by Scarnati and Tice [18] was devoted to an analysis of the building details of the device, although without any original novelty on the analysis.

More recently a very interesting paper has been published [19] that describes the use of a metal stick and a robotized device to act on the stick in a repeatable way. The conclusions of the work are quoted below:

*“(1) The revolution of the propeller is caused by the elliptical motion excited at the end of the rod*.

*(2) The vibration system can be treated as a lumped-constants system*.

*(3) Two factors of phase difference between two vibration directions exist*. *One is the shift in the resonance frequency*, *and other is waveform difference of the driving forces*.

*Furthermore*, *simulation results indicated the validity of the model composed of lumped constants*.*”*

The above state-of-the-art review suggests that the Notched Stick is commonly believed to successfully convert vibrations into rotational motion thanks to a composition of two rod movements, one along the horizontal direction and one along the vertical direction, thus resulting in an elliptical motion of the nail that drags the propeller. The aim of the present study is to show that, in spite of the common belief, the occurrence of an elliptical motion of the nail is not really necessary to induce the rotation of the propeller in this kind of device. The result is preliminarily suggested by a qualitative discussion of some simple analytical models of the toy, then experimentally demonstrated by a suitable device vibrating in a controlled way, and finally substantiated by some numerical simulations based on a more realistic analytical model. The preliminary models point out that the working principle of the device can be explained by the occurrence of parametric and/or non-parametric resonance phenomena, recognizing a mechanical analogy between the toy and a suitably excited oscillator, and removing the need for an elliptical motion of the nail. The experimental evidence is provided by tests performed, for the first time, imposing acoustic or controlled piezoelectric vibration to the stick. A further support to the conclusions comes from numerical modelling within the framework of multibody dynamics simulations. The study of the way the Notched Stick converts a vibrational stress into a rotary motion allows to speculate about the development of new technologies able to transform vibrational noise into mechanical energy.

## Features of the device and phenomena possibly involved in its running

### The device as a master-slave dynamical system

To a satisfactory degree of approximation the nail-propeller system can be regarded as a master-slave dynamical system: owing to the very small mass of the propeller, one may assume that the motion of the nail (master) is not significantly affected by the motion of the propeller (slave). This means that for the propeller the nail simply behaves as a time dependent constraint, that moves in a given way.

### Possible kinds of nail motion

At rest the nail is assumed to be placed horizontally. The nail may undergo different kinds of motion:

1. a horizontal sinusoidal motion;
2. a vertical sinusoidal motion;
3. a sinusoidal motion in a plane inclined with respect to the vertical direction, with a horizontal and a vertical component of the same frequency and with no phase difference;
4. an elliptical motion, with a horizontal component and a vertical component of the same frequency, possibly unlike amplitudes and different phases. In the case of equal amplitudes and a phase difference of ¼ a period the nail motion is circular and uniform.

In any case the frequency of the motion is that of vibration of the stick that holds the nail, induced by moving the dowel back and forth across the notches. The assumption that the two possible components of the nail motion are sinusoidal, in both the horizontal and the vertical direction, constitutes an approximation, though it seems reasonable.

### Coupling between the nail and the propeller

The contact between the moving nail and the hole at the center of the propeller may be:

- loose, like a ring hanging on a stake, or a horizontal axis hula-hoop. The clearance between the outer surface of the nail and the inner surface of the propeller hole is relatively large compared to the diameter of the nail;
- tight, which means that the external boundary of the hole basically does not slip on the nail because of the static friction forces. The rotation of the propeller takes place owing to a ball bearing set up at its center: the inner ring of the ball bearing adheres to the nail, whereas the external one moves along with the propeller.

In the first case, if along the edge of the hole the propeller remains in contact with the nail during the motion, one may assume that the propeller rolls without slipping along the edge of the nail; such a pure rolling is made possible by the static friction.

The device may be conceptualized as a horizontal axis hula-hoop in which the friction forces due to the contact between the axis and the propeller allow the rotation around the axis and may act by chance along the axis (moving the propeller back and forth) and are not balanced by the gravity as in a vertical axis hula-hoop.

### Role of gravity

The role played by gravity seems to be significant in the starting stage, the onset of the propeller motion, particularly when the oscillation amplitudes of the nail are small.

Such a role stems from the eccentric position of the propeller center of mass relative to the center of the nail, position that:

- certainly occurs in the case of a loose propeller-nail coupling;
- requires some asymmetry feature of the propeller in the case that the propeller-nail coupling is tight. If the propeller were perfectly axisymmetrical with respect to the axis of the nail, the propeller would be statically equilibrated and the effect of weight on the device dynamics would be negligible or even theoretically null.

In practice, in these conditions the propeller behaves as a physical pendulum with an oscillating point of suspension.

### Simple modelling. Tight nail-propeller coupling

**Fig 2:**
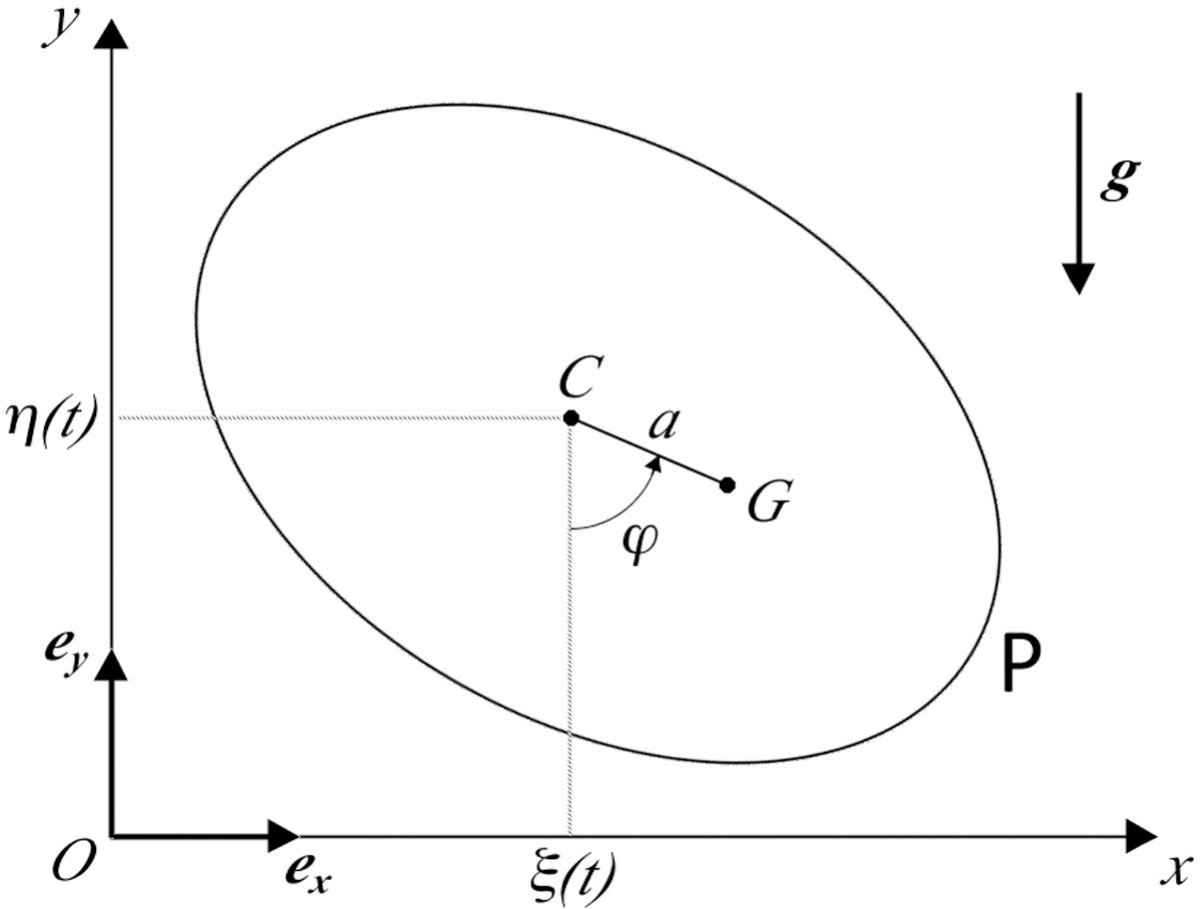
Simple model with tight nail-propeller coupling. A simple model of the device in the case of a tight nail-propeller coupling. *C* denotes the nail position in the vertical plane *Oxy*, whose coordinates *(ξ*,*η)* vary according to a given time law. G is the projection on the same plane of the center of mass of the propeller **P**. Finally, *a* stands for the (typically small) distance of *G* from the nail axis and *φ* is the rotation angle of the propeller. Constraints are assumed to be ideal, but allowance is made for energy dissipation by a viscous term $D_{\varphi }=-\beta \overset{\cdot }{\varphi }$ proportional to the angular velocity of the propeller, moving in air.

In the case of a tight coupling between the nail and the propeller a simple model of the system consists of a flat rigid body **P** that can freely rotate around a point *C* of it in a fixed plane; the point *C* is representative of the nail position and is forced to move according to an assigned time-law. One may introduce an inertial reference frame *Oxy* in the same plane, with horizontal and vertical axes *Ox* and *Oy* and unit vectors ***e***_***x***_ and ***e***_***y***_, respectively. The point *C* will move according to a law of the form:

$$C-O=\xi (t)e_{x}+\eta (t)e_{y}$$ (1)

where *ξ(t)* and *η(t)* are given functions of time. If *G* denotes the center of mass of **P**, possibly different from *C* due to slight asymmetries of the device, *a* is the distance between *G* and *C*, and *φ* stands for the angle that the segment *CG* forms with the vertical straight line drawn downwards through *C*, assuming ideal constraints the Lagrangian of the system in the reference *Oxy* takes the form:

$$L=\frac{I+ma^{2}}{2}{\dot{\varphi }}^{2}+ma(\cos\varphi \dot{\xi }+\sin\varphi \dot{\eta })\dot{\varphi }+mga\cos\varphi$$ (2)

where *I* is the moment of inertia of the plate **P** around the axis through G orthogonal to *Oxy*, *m* the mass of **P**, *g* the gravity acceleration and the dot denoted time derivative (see Fig 2).

This is the Lagrangian of an ideal holonomic system with time-dependent constraints, whose equation of motion can be written as:

$$\frac{d}{dt}\left(\frac{\partial L}{\partial \dot{\varphi }}\right)-\frac{\partial L}{\partial \varphi }=D_{\varphi }$$ (3)

i.e., explicitly:

$$(I+ma^{2})\ddot{\varphi }+mga\sin\varphi +ma(\cos\varphi \ddot{\xi }+\sin\varphi \ddot{\eta })=-\beta \dot{\varphi },$$ (4)

on having introduced a simple viscous terms $D_{\varphi }=-\beta \overset{\cdot }{\varphi }$, with friction constant *β*, to account for energy dissipation due to rotation in air.

In the case that *G* coincides with *C*, that is *a = 0*, the equation of motion reduces to $I\ddot{\varphi }=-\beta \dot{\varphi }$ and the system simply becomes a perfectly equilibrated rotor with a viscous damping; all the configurations of the plate are stable rotational equilibria and the nail motion is not effective in inducing a rotational motion of the propeller. Noticeably, gravity plays no role.

If *G* is different from *C* but gravity can be neglected, the motion is described by:

$$(I+ma^{2})\ddot{\varphi }+ma(\cos\varphi \ddot{\xi }+\sin\varphi \ddot{\eta })=-\beta \dot{\varphi }$$ (5)

and corresponds to an unbalanced rotator parametrically excited with viscous damping, already discussed in the literature as a dynamical model of hula-hoop [20].

When *G* does not coincide with *C* but gravity is significant, the full Eq 4 must be considered and the system appears as a compound pendulum parametrically excited and subjected to a viscous damping. If $\ddot{\xi }=\ddot{\eta }=0$, i.e. the system is not excited by the nail motion, there are precisely two rotational equilibria of the plate, corresponding to *φ = 0* and *φ = π*, stable and unstable respectively. As far as the possible onset of rotational motions is concerned, three remarkable cases can be distinguished according to the kind of nail motion:

- for $\ddot{\eta }=0$ but $\ddot{\xi }\neq 0$ which means that the nail *C* only moves horizontally, both the rotational equilibria of the unexcited system are deleted. Necessary condition for the onset of rotations, small oscillations around *φ = 0* may be amplified by a mechanism of ordinary resonance, since for *φ = 0* the equation of motion admits the linear approximation:
  $$(I+ma^{2})\ddot{\varphi }+\beta \dot{\varphi }+mga\varphi =-ma\ddot{\xi }$$ (6)
  and can be regarded as the equation of a damped harmonic oscillator with a forcing $-ma\ddot{\xi }$;
- if $\ddot{\eta }\neq 0$ and $\ddot{\xi }=0$ which corresponds to a purely vertical motion of the nail, the configurations *φ = 0* and *φ = π* are still rotational equilibria of the system. However the configuration *φ = 0* may become unstable owing to phenomena of parametric resonance [21], because the motion is governed by an exact equation of Hill’s type with damping:
  $$(I+ma^{2})\ddot{\varphi }+ma(g+\ddot{\eta })\sin\varphi =-\beta \dot{\varphi }.$$ (7)
  In particular, for *η = ε cosωt*, with *ε* and *ω* some positive constants, the result is a Mathieu equation with damping;
- finally, whenever both $\ddot{\xi }$ and $\ddot{\eta }$ are nonzero, thus describing the case where the nail oscillates in the horizontal as well as the vertical direction, the rotational equilibria *φ = 0* and *φ = π* of the unexcited system disappear and the onset of the rotation may arise from both kinds (parametric and ordinary) of resonance.

### Simple modelling. Loose nail-propeller coupling

In the case where the coupling between the nail and propeller is loose, the propeller can be modelled as a possibly inhomogeneous circular ring *Γ*, of mass *m*, that can roll without slipping, in a fixed plane, on the outer edge of a circular disk *D* representative of the nail. The disk undergoes an assigned, purely translational motion which corresponds to the imposed motion of the nail. Such a translational motion can be simply described by the motion of the centre A of the disk.

**Fig 3:**
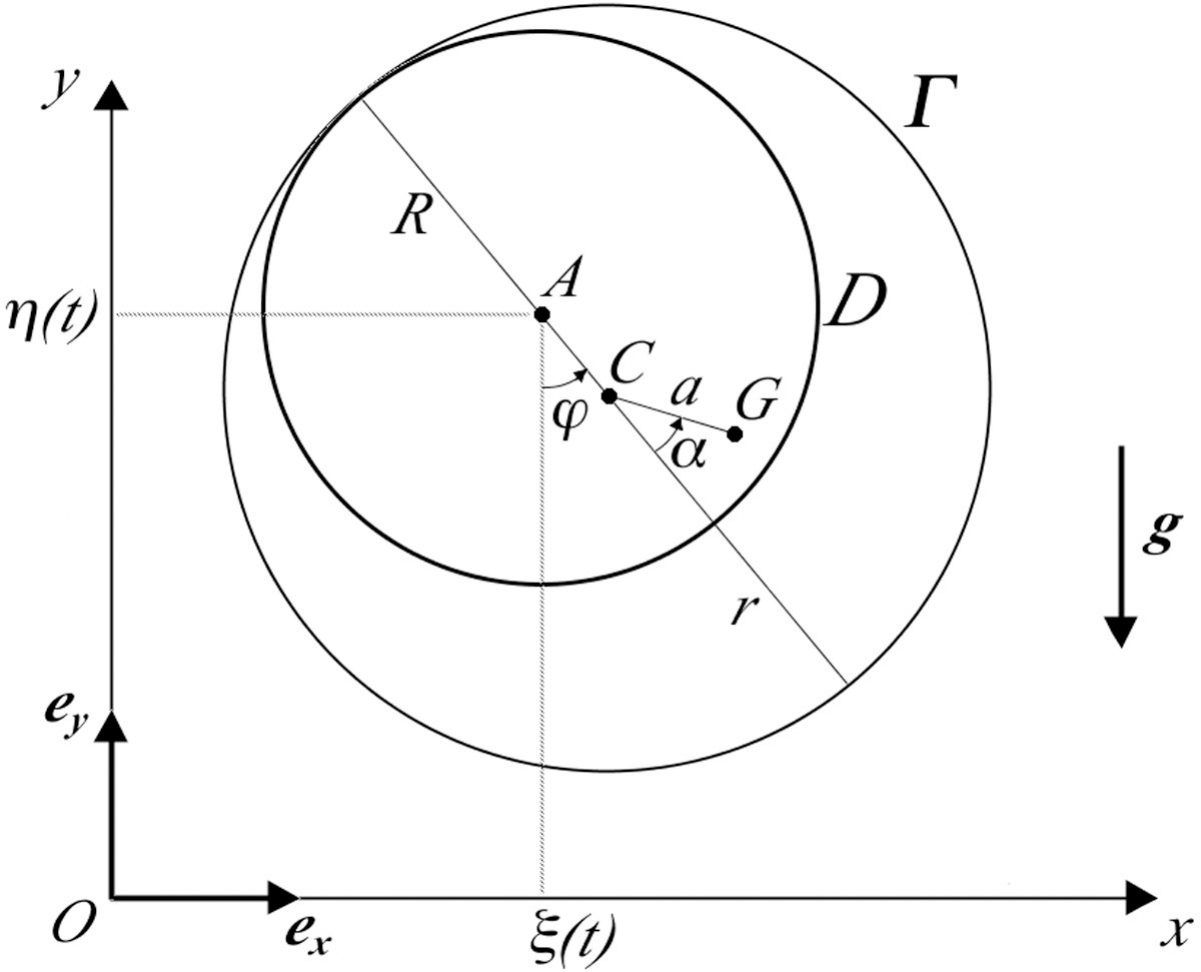
Simple model with loose nail-propeller coupling. A light pitchless propeller loosely mounted at the end of the rod by a nail to rotate freely. Another model of the device more appropriate for the case of a loose nail-propeller coupling. In the vertical plane *Oxy* the nail is represented by a rigid disk *D* of center *A* and radius *R*, animated by a purely translational motion whose description is given in terms of the varying coordinates *ξ(t)*, *η(t)* of *A*. The inner edge of the propeller hole, represented by the rigid circular ring *Γ* of radius *r* and center *C*, is assumed to roll without slipping on the outer profile of the nail *D*. Pure rolling requires static friction between *Γ* and *D*, but it does not invalidate the assumption of ideal constraints. The moment of inertia of the propeller with respect to the axis passing through its center of mass and orthogonal to *Oxy* is supposed to be known. The orthogonal projection *G* of the center of mass on the plane *Oxy* may not coincide with the center *C* of the ring *Γ*, as described by the distance *a* and the angle *α*. The propeller rotation is parametrized by the angle *φ*.

Denoted with *R* the radius of the disk, and with *r* (*r>R*) and *C* the radius and the center of the ring *Γ*, respectively, the angular velocity of *Γ* can be easily expressed in terms of the angle *φ* that the segment from *A* to *C* forms with the vertical straight-line drawn through *A* downwards (see Fig 3):

$$\omega_{\Gamma }=\frac{r-R}{r}\dot{\varphi }$$ (8)

and is a vector orthogonal to the plane of the motion. The center of mass *G* of the ring may possibly differ from the centre *C* due to slight asymmetries of the propeller, and its position relative to *G* in the rest frame of the ring can be specified by the constant distance *a = CG* and the constant angle *α* shown in Fig 3.

As before, an inertial reference frame *Oxy* may be introduced in the plane of the motion, with horizontal and vertical axes *Ox* and *Oy* and unit vectors ***e***_***x***_ and ***e***_***y***_, respectively. The centre *A* of the disk will move according to a given time law of the form:

$$A-O=\xi (t)e_{x}+\eta (t)e_{y}$$ (9)

with appropriate functions of time *ξ(t)* and *η(t)*. If the constraints are assumed to be ideal, the Lagrangian of the system in the reference *Oxy* is given by:

$$\begin{array}{l} L=\frac{m}{2}\left[{\left(r-R\right)}^{2}+a^{2}+2(r-R)a\cos\alpha \right]{\dot{\varphi }}^{2}+\frac{I}{2}\frac{{\left(r-R\right)}^{2}}{r^{2}}{\dot{\varphi }}^{2}+m\dot{\xi }\dot{\varphi }\left[\left(r-R)\cos\varphi +a\cos(\varphi +\alpha \right)\right]+\\ +m\dot{\eta }\dot{\varphi }\left[\left(r-R)\sin\varphi +a\sin(\varphi +\alpha \right)\right]+mg\left[\left(r-R)\cos\varphi +a\cos(\varphi +\alpha \right)\right] \end{array}$$ (10)

and the equation of motion takes the form:

$$\begin{array}{l} m\left[{\left(r-R\right)}^{2}+a^{2}+2(r-R)a\cos\alpha \right]\ddot{\varphi }+I\frac{{\left(r-R\right)}^{2}}{r^{2}}\ddot{\varphi }+m\ddot{\xi }\left[\left(r-R)\cos\varphi +a\cos(\varphi +\alpha \right)\right]+\\ +m\ddot{\eta }\left[\left(r-R)\sin\varphi +a\sin(\varphi +\alpha \right)\right]+mg\left[\left(r-R)\sin\varphi +a\sin(\varphi +\alpha \right)\right]=-\beta \dot{\varphi }, \end{array}$$ (11)

where *I* denotes the moment of inertia of the ring relative to its centre of mass *G* and, as before, allowance is made for energy dissipation by means of a viscous term $D_{\varphi }=-\beta \dot{\varphi }$.

If the disk is at rest, so that $\ddot{\xi }=0$ and $\ddot{\eta }=0$ (fixed nail), the two rotational equilibria of the ring *Γ* are derived from the obvious equation (r−R)sinφ+asin(φ+α)=0. Such equilibria persist if $\ddot{\xi }=0$ and $\ddot{\eta }\neq 0$ (purely vertical motion of the disk), and the amplification of small motions around the stable equilibrium should be attributed to processes of parametric resonance able to destabilize it. In contrast, rotational equilibria are removed in the case of purely horizontal motion of the disk (i.e., $\ddot{\xi }\neq 0$ and $\ddot{\eta }=0$) and the possible growth of oscillation amplitude is due to ordinary resonance. In the general setting ($\ddot{\xi }\neq 0$ and $\ddot{\eta }\neq 0$), parametric and ordinary resonance processes are expected to coexist.

Noticeably, in the typical case where *α = 0* and/or *a = 0* Eq 11 takes the same form of Eq 4 already derived for the previous model of tight nail-propeller coupling.

If additionally the effect of gravity is small and the oscillations of the disk can be assumed harmonic, another typical condition for common Notched Stick devices, the existence of almost uniform and stable rotational motions can be analysed and justified [22].

The following Table 1 resumes all the cases.

**Table 1:** Resume of the basic nail-propeller relation.

Loose coupling		Tight coupling	
Always gravity role		Gravity role only if asymmetry is present	
Equation of motion (11)		Equation of motion (4)	
If *a* = 0 and/or *α = 0*: equation of motion (11) takes the same form of Eq (4)		*If a* = 0: perfectly equilibrated rotor with viscous damping; no gravity role; no rotation	
		*If a* ≠ 0 and negligible gravity: asymmetric rotor with viscous damping; possible rotation	
Onset of rotation	$\ddot{\eta }$ = 0 $\ddot{\xi }≭$0 *C* moves horizontally; ordinary resonance	Onset of rotation	$\ddot{\eta }$ = 0 $\ddot{\xi }≭$0 *C* moves horizontally; ordinary resonance
	$\ddot{\eta }\neq$0 $\ddot{\xi }=$0 *C* moves vertically; parametric resonance		$\ddot{\eta }\neq$0 $\ddot{\xi }=$0 *C* moves vertically; parametric resonance
	$\ddot{\eta }\neq$0 $\ddot{\xi }≭$0 *C* moves in both directions; both resonances		$\ddot{\eta \neq }$0 $\ddot{\xi }≭$0 *C* moves in both directions; both resonances

A resume of all the possible settings of the simple dynamical model of the device. For the meaning of all symbols see the text.

### Effect of the nail motion

As a consequence of the previous discussion, the effect of the nail motion on the propeller can be easily described in a qualitative way.

If the propeller is tightly connected to the nail and turns out to be perfectly axisymmetrical with respect to the axis of the nail (the propeller center of mass lies on the symmetry axis of the nail), the nail motion does not trasfer any significant rotation to the propeller, but possible small effects due to friction forces.

In contrast, if the propeller center of mass is not aligned with the nail axis, the propeller behaves as a parametrically excited (and damped) compound pendulum and the following situations may occur:

1. if the nail only oscillates in the vertical direction, the trivial equilibrium position of the propeller survives, but it could be made unstable by a process of parametric resonance;
2. when the nail only oscillates in the horizontal direction, the trivial equilibrium position is removed and the oscillation amplitudes of the propeller may grow, up to the possible onset of a rotation, as a result of a process of ordinary resonance;
3. if the nail oscillates along an intermediate direction between the horizontal one and the vertical one, both the effects previously described take place;
4. if, finally, the nail describes an elliptical motion, as a composition of two harmonic motions of the same frequency, arbitrary amplitudes and a phase difference which is not an integer multiple of *π*, a condition similar to case (3) occurs.

Moreover, a similar behaviour would be observed also in the case where the weight of the propeller were completely negligible. In that case, however, all the configurations would be of rotational equilibrium in the absence of nail motion.

Nevertheless, introducing an oscillation of the nail along a given direction would destroy all the positions of rotational equilibrium but those where the propeller center of mass is aligned with the direction of the nail oscillation; such configurations, however, could be (or could not be) unstable as a consequence of parametric resonance phenomena and give rise to a rotational motion of the device [22].

The possible oscillation of the nail along an elliptical path would remove all the positions of rotational equilibrium, thus allowing the onset of rotations of the propeller by analogous mechanisms.

Generally speaking, the onset of a rotational motion of the propeller DOES NOT NECESSARILY REQUIRE that the nail must follow an elliplical motion, but it may occur also in the case of a purely linear motion of the nail along a fixed direction: along the vertical (rotation triggered by parametric resonance), along the horizontal (rotation induced by ordinary resonance), or along an intermediate direction (rotation induced by a combination of both kinds of resonance).

The previous models, although useful to illustrate qualitatively the possible onset of a rotational motion of the propeller as a consequence of a nail oscillation along a straight-line, suffer however of a considerable level of approximation to be completely useful in the quantitative description of the real device. In the case of a loose propeller-nail coupling, and particularly in the presence of very strong vibrations of the nail, one may expect temporary losses of contact and subsequent collisions between the propeller and the nail, involving impulse forces that would make the dynamics of the device hardly predictable, particularly in the initial stage of onset of the possible rotation. Another phenomenon to take into account is the possible sliding of the propeller hole edge on the outer surface of the nail, which makes the system eventually affected by dynamic friction forces. Last, but certainly not least, the assumption that the motion of the propeller takes place in a fixed vertical plane is typically unrealistic for a practical device, since the propeller may slide back and forth along the nail surface, parallel to the nail axis.

The above remarks suggested two lines of investigation:

1. the design and implementation of an appropriate device, to carry out experimental observations under conditions as controlled as possible;
2. the development of a more realistic (and complex) numerical model to reproduce, as far as possible, the results of the experiments. Obviously, as we will see, the price to pay was a growth of the number of degrees of freedom and an adequate modeling of impulse and sliding friction forces.

## Materials and experimental methods

### Materials

The used sticks were made of chestnut or beech, the rotating part of raft wood or polyurethane. The cross-section of sticks was 9x9 mm or 9x21mm. The rotating axis simply consisted of a slightly conic nail of mean diameter (1.64mm) fixed on the top of the stick. The size of different specimens was chosen as close as possible.

In order to impose a controlled vibration to the stick a loudspeaker and a piezoelectrical device (piezo) were exploited. These devices are currently used in our laboratory for experiments in wettability and partly described in previous literature [23,24]; the piezo movement PI 601 with a 505 PI power source moves along one direction only with a maximum elongation of 300 μm. For both devices a self-developed Labview software has been used to manage the vibration through a computer card or an external amplifier. In the case of the piezo the effective exciting voltage was checked through a parallel electric connection (manual feedback control).

The length of the stick was evaluated with respect to the vibrating support, with the same length protruding from the support and fixed with a very rigid rubber band in the case of the loudspeaker, or with a metallic constraint blocked with screws in the case of the piezo support.

It is noticeable that to simulate the presence or the absence of a mechanical backlash between the rotation axis and the rotating part of the stick we have used a microbearing on which the rotating propeller has been mounted; the microbearing could be fixed to the nail or not. The presence of a microbearing fixed to the nail corresponded to the absence of the backlash, because the propeller was fixed to the exterior of the bearing. In the absence of a microbearing stuck to the nail, the rotation of the propeller around the nail was affected by the microbearing inner diameter, inevitably larger than the nail diameter and thus associated to a backlash of a fraction of a millimeter. In this second case the presence of a microbearing avoids that the soft material of the propeller could be damaged or modified by the experimental procedure and keeps constant the backlash size.

All the experiments have been carried out on an antivibrating table. This condition is very important: runs performed on a common laboratory table do not give the described results. This is probably due to the fact that, in absence of an antivibrating support, the vibrations generated by the loudspeaker or by the piezo are in fact dissipated by the support. The experiment has been mounted on a Newport VH 3030-OPT antivibration table.

### Experimental method

The apparatus described above has been used in different ways; the sinusoidal vibration has been imposed to the stick put transversally on the loudspeaker or fixed to the piezo base.

The main difference between these two vibration sources is that in the case of the loudspeaker some vibration on the horizontal axis cannot be avoided, even if the main contribution is reasonably in the vertical direction. In contrast, for the piezo source one can impose a purely vertical or horizontal vibration, or even at a 45° inclined direction, combining the effect of horizontal and vertical vibration (in phase).

The experiments have been performed using a propeller without or with backlash, the first case corresponding to the use of the microbearing fixed to the nail, and the second to a propeller without the microbearing or, better, endowed with a microbearing not fixed to the nail.

In all these cases short movies have been recorded from which it was possible to extract some useful information such as the rotation direction, the speed and acceleration of the propeller and those of the nail head.

To collect the movies an EXILIM EX-FH25 Casio camera has been used, able to collect movies until 1000 fps. This kind of camera is very cheap and thus has some intrinsic limitation, reducing the pixel number of the image at the highest speed. The reduced size allowed however to capture the system image with an acceptably high resolution. The Casio Exilim FH-25 has a powerful macro objective; the movies have been taken at different distances from the rotating propeller; in the case of 1000 frames/sec images the pixel number is only 228x64. In the movies used for the calculation of rotation frequency the resolution was of about 3pixel/mm, but to detect the nail oscillation the pixel/mm ratio was modified to at least 10pixel/mm; therefore the final image resolution is of the order of 0.1mm. The minimum focus distance in supermacro mode is 1cm only, thus probably it is possible to achieve even a better pixel/mm ratio than that obtained in the present study.

**Fig 4:**
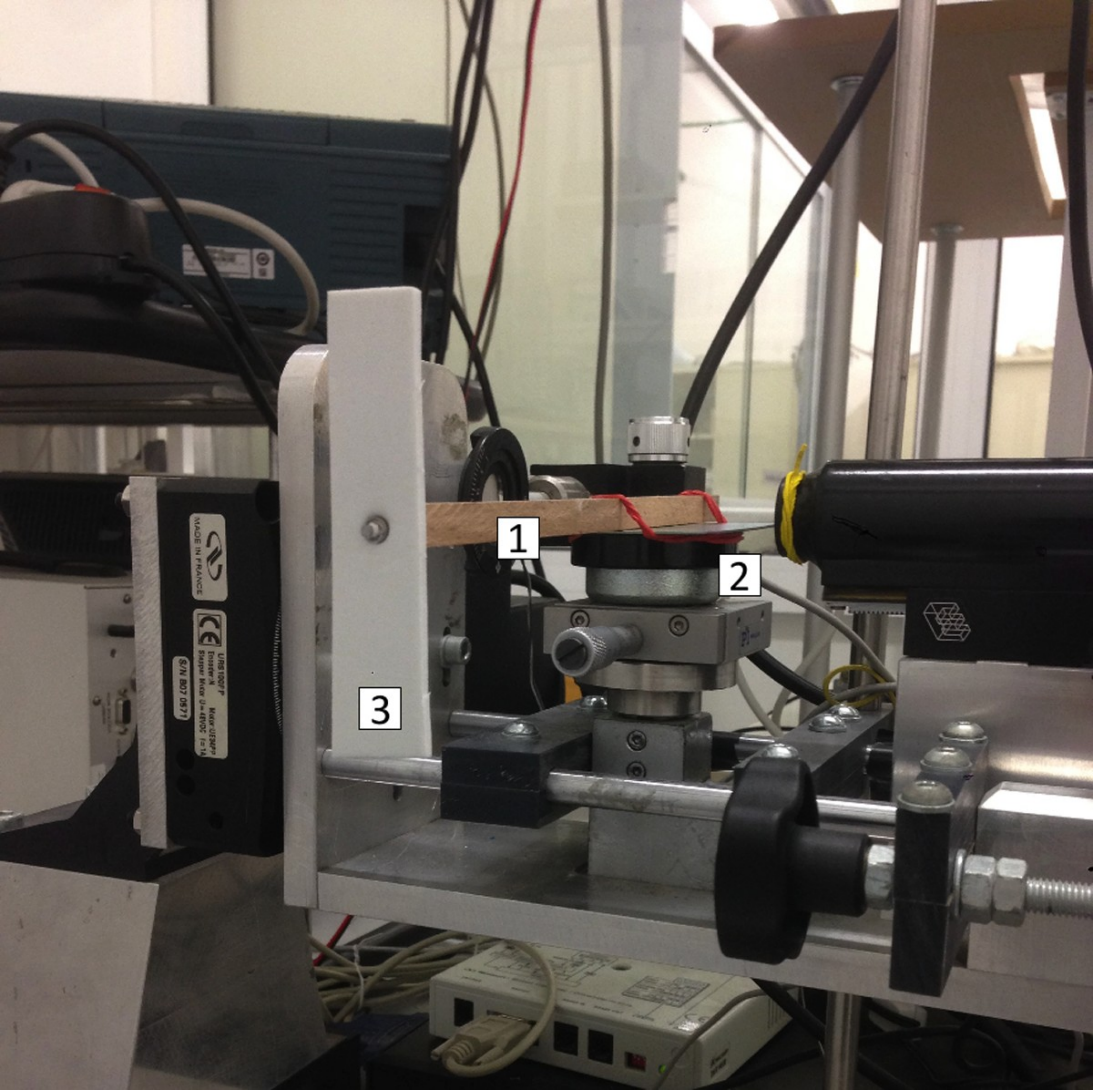
Experimental setup. The experimental setup of the system, with the notched stick (1) mounted on the loudspeaker (2) and endowed with a polyurethane propeller (3).

A picture of the experimental device is shown in Fig 4.

## Experimental results

**Fig 5:**
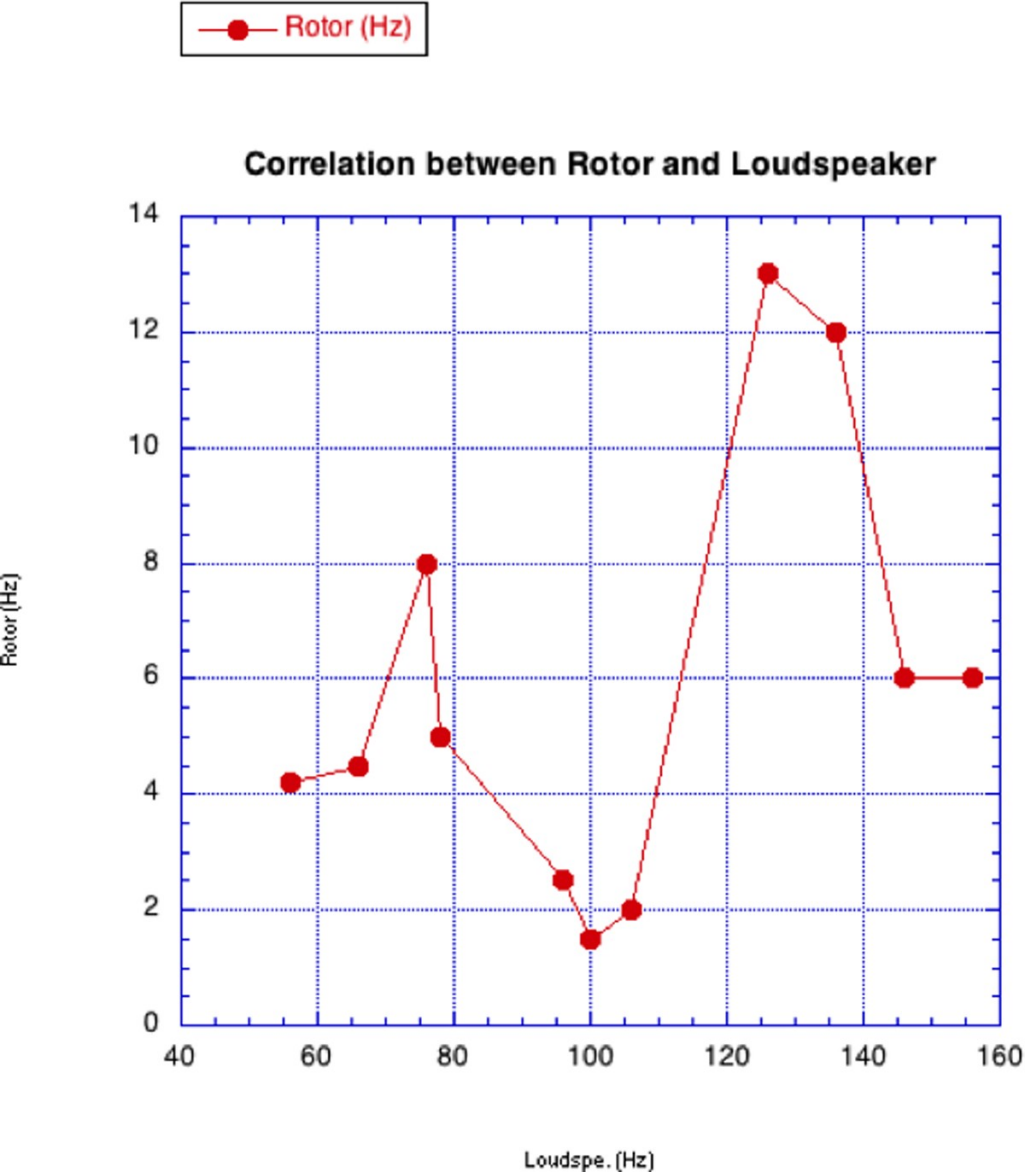
Experimental results for the loudspeaker vibration source. Rotor frequency vs. loudspeaker frequency for a notched stick mounted on a loudspeaker and with a free moving microbearing.

In a first stage of the experiments the vibration-to-rotation tests have been carried out using a loudspeaker as a generator and without any bearing. It was apparent that some excitation frequencies are more efficient than others in the transformation of vibrational to rotational motion. It seemed useful, however, to reduce the oscillation amplitude of the propeller by means of a microbearing NOT fixed on the nail, simply to impose a fixed backlash between the nail and the bearing with a very small lateral oscillation. In these conditions it was possible to obtain the results shown in Fig 5. The Notched Stick was fixed to the loudspeaker through a very tight, although elastic connection.

The plot shows the number of rotations per second versus the strain frequency (red line) on the horizontal axis. It is possible to detect a couple of resonance frequencies, whose value is about 75 Hz and 130 Hz, probably related by a harmonic correlation.

These experiments showed however that it was difficult to exclude a horizontal component due to the relative weakness of the elastic bonding and to the loudspeaker behaviour. For this reason the experiments have been repeated using the piezoelectric generator as a vibration source; in this case it was possible to induce a purely vertical strain of the stick.

**Fig 6:**
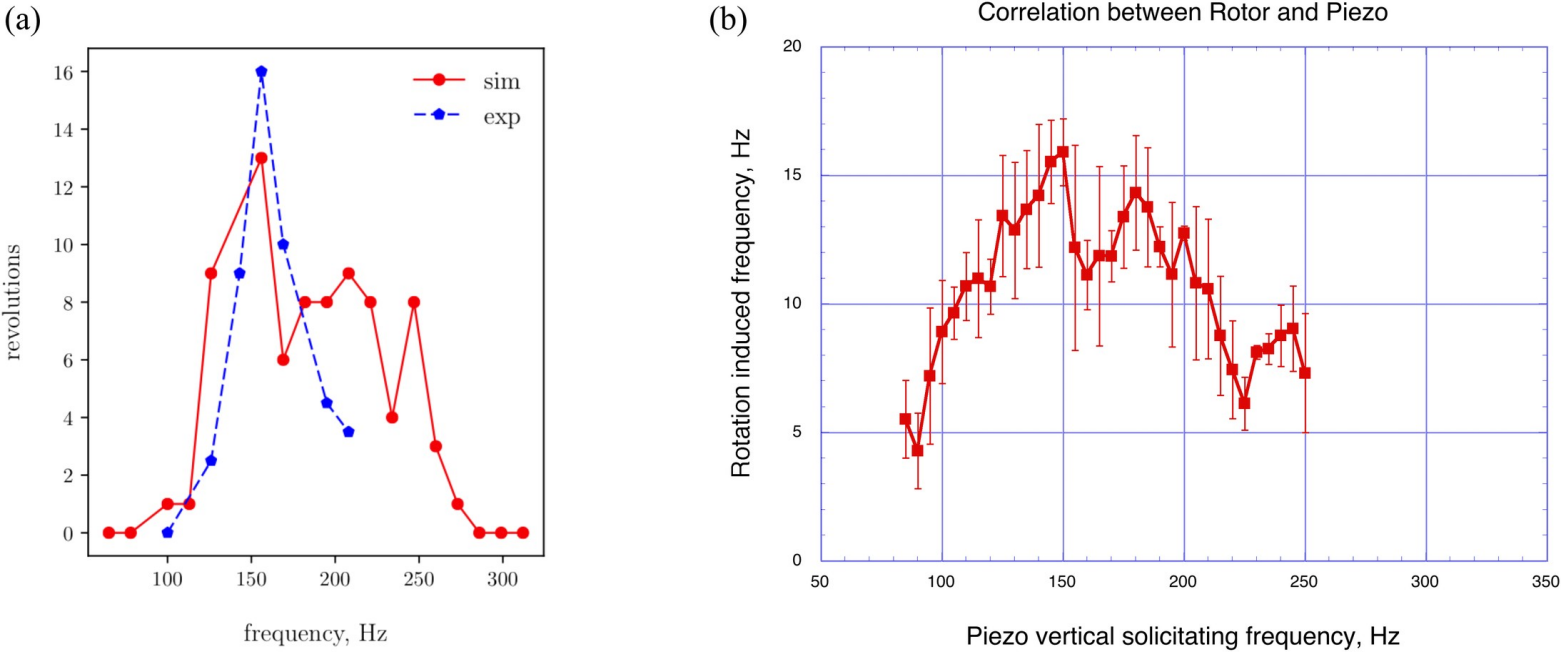
Experimental results for the piezo vibration source and comparison with simulations. **(a**) Comparison between some experimental and simulated data for a Notched Stick with a plastic rotor on a free moving microbearing mounted on a piezo vibratory device. Revolution frequency of the propeller as a function of excitation frequency. Each point corresponds to a single run of the experiment or the numerical simulation. The range of excitation frequencies at which the propeller rotates is similar for experimental and simulated tests. (**b)** Experimental data for a Notched Stick with a plastic rotor on a free moving microbearing mounted on a piezo vibratory device. Revolution frequency of the propeller as a function of excitation frequency in a larger interval than in Fig 6A. Repeated experiments allow to estimate the standard deviation of rotation frequency; the error on the imposed piezo frequency is negligible. The use of a piezo vibration source makes it possible a better control of the vibration plane. The data confirm the frequency peak at about 156 Hz and show a qualitative agreement with the simulated results of Fig 6A. Discrepancies in the experimental results of Fig 6A and 6B are probably due to the circumstance that, for technical reasons, between the two sets of measurements the apparatus had to be dismounted and the restored again (re-use of the antivibrating table for other purposes). The unavoidable aging of wood and polyurethane components, induced by the many operations carried out on the device, may also have played a minor role.

The effect observed on the same device with the same protruding length and a very rigid mechanical blocking is illustrated in Fig 6A. In this condition (blue line) the frequency of 156Hz has been found as the main resonance frequency.

In order to improve statistics, compute standard deviations and check repeatability, the same measurements have been carried out four times, also in a larger interval of excitation frequencies than that in Fig 6A. The results are shown in Fig 6B, where vertical error bars represent one standard deviation of uncertainty, while the error on the imposed piezo frequency turns out to be negligible. Notice the similarity with the simulated results of Fig 6A.

The same experiments has been repeated by two different methods: applying the mechanical strain along the horizontal axis and eliminating the backlash, with the fixation of the microbearing on the nail (same diameter). The resulting effect is that while the horizontal strain does not change the result in a significant way, the elimination of the backlash prevents the rotation; the propeller simply moves erratically in both directions without any regular rotation.

**Fig 7:**
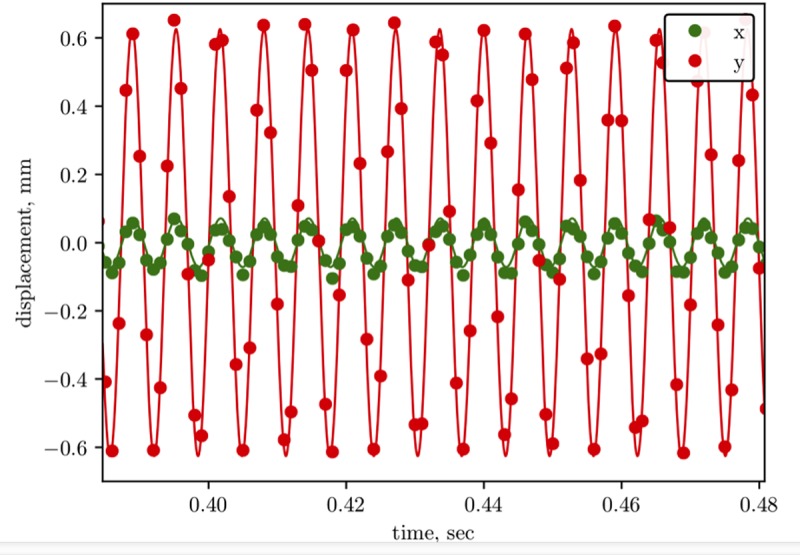
Displacement of the nail head. Displacement of the center of the nail head measured from the recording of an experiment performed stimulating the Notched Stick through the piezoelectrical device applying 156Hz frequency. Dots are the measured data and solid lines the sinusoidal fit.

Videos have been used to check if the motion of the nail is mainly vertical, as shown in Fig 7.

At this point we have decided to apply the mechanical strain along an intermediate direction, so that we may consider the result as a combination of a vertical and a horizontal excitation; this has a positive effect in the presence of a backlash, but yields no rotation effect in the case where backlash is absent.

### Nail head motion tracking

In order to understand the effect of the vibration imposed by the loudspeaker and the piezoelectric devices on the motion of the Notched Stick components, experiments performed at different excitation frequencies were recorded by means of the previously described high-speed camera. In particular, the displacement of the head of the nail both along the vertical (y) and the horizontal (x) axes was measured from collected movies by means of a tracking algorithm exploiting OpenCV [25] routines. More precisely, a contrast-based criterion was used to define the nail head and the position of its center of mass was recorded at each frame.

For the experiment performed adopting the piezo vibration source and a strain frequency of 156Hz, Fig 7 reports a portion of the resulting nail head trajectory fitted by a sinusoidal functions. Although the piezo apparatus should in principle transfer the vibration along a single axis, a displacement of the nail head along both the x and the y direction was measured. However, it should be pointed out that the x component is probably due to the not perfectly rigid connection between the piezo and the Notched Stick devices and that it shows a much smaller amplitude than the y one, thereby not strongly affecting the motion.

## The multibody dynamics model

### Materials and methods

In addition to the experiments, multibody dynamics simulations of the Notched Stick were performed within the framework of the Msc.Adams software [26]. This numerical simulation technique is a useful tool for the investigation and the design of mechanical systems comprising of several moving bodies, providing with a clear picture of their dynamic and kinematic response under different conditions and allowing to test any possible configuration [27].

**Fig 8:**
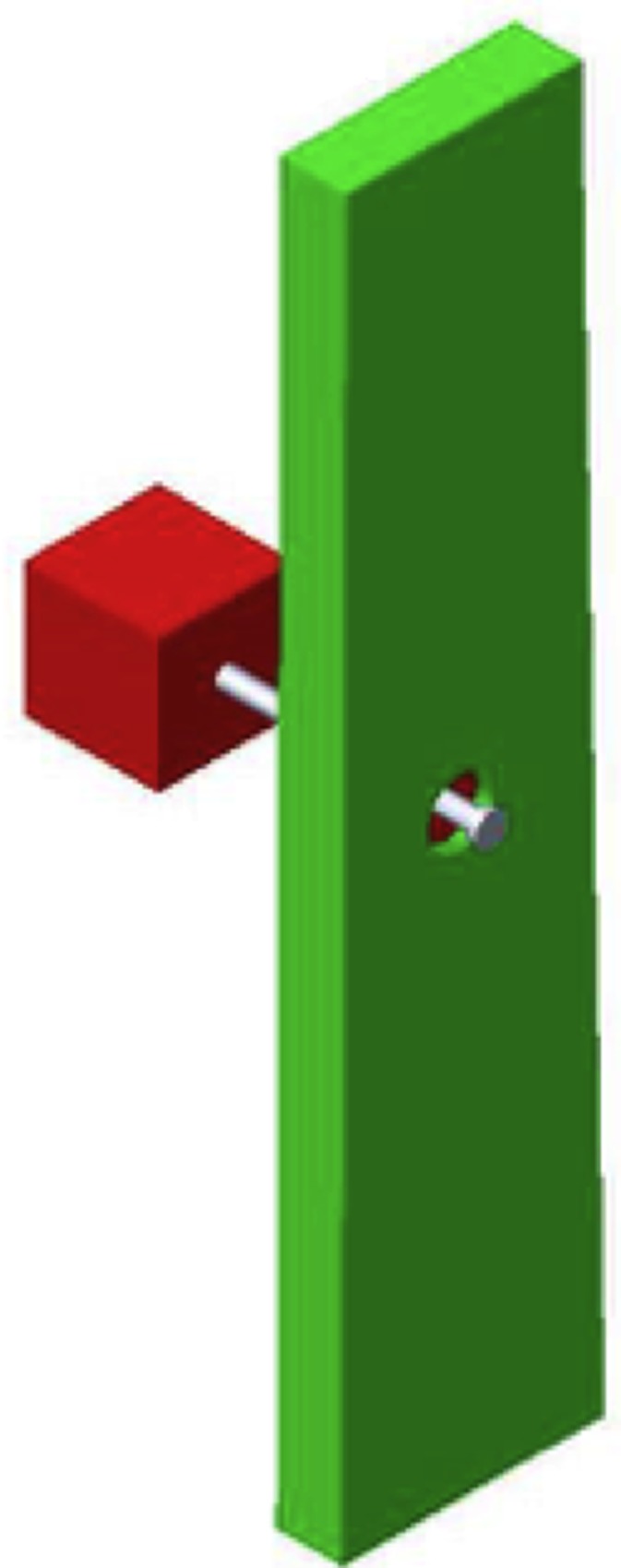
Geometrical model of the Notched Stick for simulations. The geometrical model of the Notched Stick adopted in multibody dynamics simulations consisting of the propeller, the nail, the microbearing and an additional part, preventing the propeller disengagement.

In simulations the Notched Stick was reduced to the nail, the propeller and the microbearing, as shown in Fig 8. Each element was modeled as a rigid body with geometrical sizes and physical properties of the experimentally-tested prototype, described in the Materials section. A small cubical block with wood characteristics, mimicking the terminal portion of the stick, was also added to prevent the propeller to slide out of the nail during the motion.

The propeller and the bearing were rigidly joined while the tip of the nail was fixed to the ground reference frame through a cylindrical shaft.

The interactions among the Notched Stick components were modeled by adding a contact force, *F*_*c*_, to the equations of motion upon the occurrence of a collision (continuous contact model, [28,29]). Among the possible formulations of *F*_*c*_, the MSC.Adams hard-coded *impact function* [26] was exploited in this work, expressing this force as a combination of a non-linear spring in parallel with a damper

$$F_{c}=k\;u^{n}+c\;u^{'},$$ (12)

where *u* and *u*^*'*^ are the relative displacement and velocity of the interacting bodies, *k* the spring generalised stiffness parameter and *n* the non-linear power exponent. *c* denotes instead the damping coefficient and, to properly represent the energy dissipation rate and prevent numerical instability, it varies gradually from 0 to a maximum value *c*_*max*_ depending on the relative displacement of the interacting bodies, with *c*_*max*_ applied when u ≥ u^*^.

Parameters *c*_*max*_ and u^*^, as well as *k* and *n* are to be defined by the user and several strategies can be adopted for their assessment. Particularly, in this work, *c*_*max*_ and u^*^ were tuned so to get a match between the number of revolutions of the propeller measured in simulations and in experiments performed with the piezoelectric oscillator for a 156 Hz frequency.

An estimate to the scale of the stiffness parameter *k* was deduced instead from the Hertz theory of contact (assuming *n* = 3/2) [30]. Also friction at the contact location was accounted for by adopting the MSC.Adams hard-coded formulation of the Coulomb model [26], with the friction coefficient smoothly varying from the static, μ_s_, to the dynamic friction value, μ_d_, as a function of the tangential relative velocity of the colliding bodies [31].

The interaction between the nail and the bearing, both made of steel, was modeled assuming *c*_*max*_ = 0.6 kg/s, u^*^ = 0.01 mm, *k* = 1e^5^ N/mm^3/2^, μ_s_ = 0.74, μ_d_ = 0.6, with the latter two coefficients corresponding to the steel-steel dry friction values suggested by literature [32]. For the contact between the propeller and the wood-like block, parameters *c*_*max*_ = 5 kg/s, u^*^ = 0.01 mm, *k* = 5e^3^ N/mm^3/2^ were imposed.

The Lagrangian equations of motion of each modeled body were solved using the Hilber-Hughes-Taylor (HHT, [33,34]) integrator with automatic step tuning and maximum numerical error of 10^−6^.

To simulate the motion of the Notched Stick under piezoelectric vibration, the displacement of the nail head was imposed (to the nail head centre) by the sinusoidal law

y=Asin(ωt), (13)

mimicking the oscillation along the vertical axis of the nail that is induced in the experiments (see the Experimental results section) by the vibration transmitted from the stick. Amplitude, *A*, and frequency, *ω*, were deduced by fitting the above equation to the displacement measured from the high-speed video recordings of the 156 Hz experiment by tracking the motion of the nail head (see Fig 7).

Also the motion given by the loudspeaker can in principle be simulated by applying simultaneously two sinusoidal displacements to the nail head, imposing the oscillation of the nail along both the vertical and the horizontal axes. However, since the vertical component has generally a predominant influence on the motion of the propeller, in this work numerical simulations focus on the simpler case of a single vertical oscillation.

For this nail motion condition, simulations lasting 8s were performed varying the excitation frequency and compared with experimental results. The model was then further exploited to test the effect of the backlash between the microbearing and the nail, Δ*r*, by gradually modifying the microbearing radius, *r*, between the two limit conditions *r* = 0 (no microbearing) and *r* = *r*_*nail*_ (no backlash).

### Numerical results

Previously described numerical simulations and experimental tests performed with the piezoelectrical vibration source provided the revolution frequency of the Notched Stick propeller as a function of the tested oscillation frequencies. Fig 6A compares the results and shows a certain resemblance between the two data sets, both in terms of trend and average number of revolutions.

**Fig 9:**
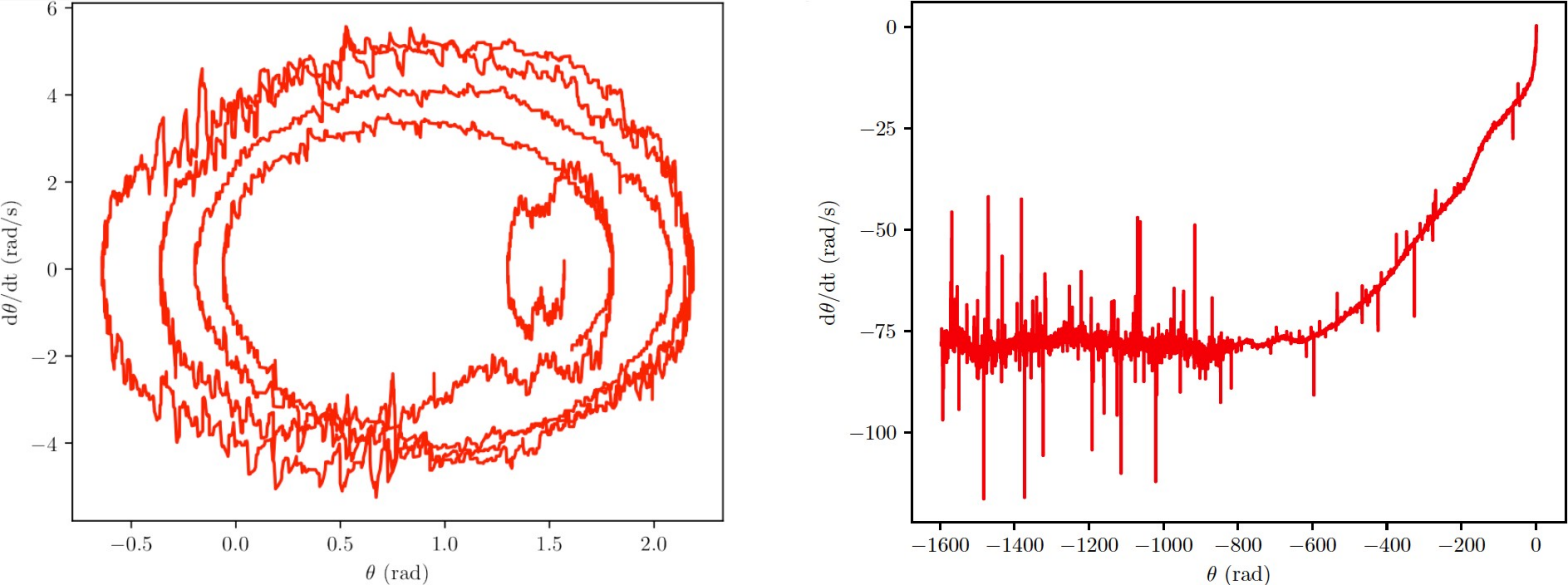
Phase portraits. Phase portrait of the propeller. As in the case of the simple pendulum, when the propeller does not rotate, e.g. at 65 Hz (left), the diagram shows the trend of a closed curve. In contrast, the trend of the diagram at 156 Hz is that of an open curve (right), due to the revolution of the propeller; the final stage of the run appears rather noisy, probably owing to nail/propeller shocks and relative slip, that sometimes may suddenly enhance friction forces and angular speed variations. Unfortunately, the backlash between the nail and the propeller turns out to be necessary to the onset of rotations, but it also makes the dynamics of the system far form being trivial.

Additionally, simulations provided the angular displacement, θ, and the angular velocity, dθ/dt, of the propeller. The plot of these quantities is depicted in Fig 9 for two different excitation frequencies and some affinities with the phase portrait of the simple pendulum should be pointed out. Indeed, when the propeller oscillates but does not rotate–e.g. at 65 Hz (Fig 9, left)–a closed displacement-velocity diagram (phase plot) is obtained. Instead, as the spinning starts–e.g. at 156 Hz (Fig 9, right)–an open phase diagram is derived.

Besides the effect of the excitation frequency, modelling was further used to analyse the influence of the backlash between the bearing and the nail radii, Δr, a variable that previously described experiments suggested to be crucial for the revolution of the propeller. Bearings with internal radius between 0.82 mm (bearing radius equal to the nail radius, Δr = 0, no backlash) and 1.25 mm were introduced in the Notched Stick model and each of them was tested at 126, 143, 156, 169 Hz. Primarily, simulations enforced the experimental observation that Δr = 0 prevents any rotational motion of the propeller. For all the tested frequencies, revolution was then shown to take place for Δr ranging between 0.09 and 0.28 mm.

Simulation outputs confirmed therefore that the Notched Stick requires a loose connection between the nail and the propeller to convert the transmitted vibration into spinning of the propeller. Although imperfect, the propeller of our experimental device is relatively well-balanced: its axis of symmetry passes through its center of mass. If no backlash were present, the center of mass of the propeller would almost coincide with the center of rotation of the propeller about the center of the nail; the forces applied to the propeller (friction and inertial forces in the reference frame where the nail head is at rest) would have no way to yield a significant moment on the propeller, that hardly could be forced to rotation. However, results highlighted that not every backlash ensures the revolution and provided a range of eligible Δr values for the investigated Notched Stick configuration.

It is finally worth noting that for the Δr = 0 case, simulations were performed by applying not only the motion of the nail along the vertical axis (defined by Eq 13), but also (i) a single sinusoidal oscillation of the nail along the horizontal axis and (ii) a vertical and a horizontal vibration simultaneously. Nevertheless, for all these cases no rotation of the propeller was observed, thus strengthening the conclusion that a backlash is necessary to develop such a kind of motion.

## Discussion

As shown by the scientific literature on the Notched Stick discussed in the Introduction section, two main mental models have been developed to explain how the device works: (a) combination of harmonic motions and (b) hula hoop description. Moreover no experimental approach has been developed different from the mere manual or robotized use of the original device.

We stress that the term vibrot is used here in a broader sense, i.e. it is intended to mean generically a “device able to convert linear oscillatory motion into rotational motion”, never mind the basic mechanism responsible for such a conversion, while in a more strict sense a vibrot is a device specifically designed to this purpose, in order to obtain: (1) a preferred one-sided orientation of the rotational motion, and (2) a reasonable (although however low) efficiency in the conversion. Nevertheless the set of experiments, whose results are shown in Fig 6B, has allowed to confirm that the Notched Stick may at least partially satisfy the requirement (1) of the “strict” definition, since in most cases (80–90%) the rotation was counterclockwise (front view), in 5 to 10% cases clockwise and in less than 5% changed its sense during the experiment.

We have introduced a simpler experimental but well repeatable method which is based on the use of a common vibration generator (a loudspeaker or a piezoelectric oscillator). This different experimental approach probably simplifies the phenomenon but allows to focus our attention on the intrinsic mechanism of transformation of the original vibration into a rotation.

The device may certainly be considered as a pendulum, whose support is not completely fixed but may move in a well defined zone (delimited by the backlash size) along the vertical or horizontal direction.

In general a pendulum may acquire energy and overcome the upper dead point approaching a rotation condition and this holds true also for the Notched Stick. However, the possibility to vary periodically the position of the support along the two directions may explain the onset of rotational motion in terms of parametric and non-parametric resonance. This same possibility introduces the alternative description in terms of a horizontal hula-hoop. The classical hula-hoop rotates about a vertical link (commonly the human body or a part of it) and its movement can be described as resulting from the balance of the weight of the hula-hoop and the friction force due to the contact with the body. In this way the hula-hoop may rotate, the rotation generates the friction and the friction opposes to the weight; the motion has ben interpreted as a combination of parametric and non-parametric resonance, as discussed in a classical paper [34].

What happens if the hula-hoop rotates about a horizontal link (i.e., an arm)? Such a problem has been analysed in literature, even if the most interesting papers are in Japanese [35]; however this provides an equivalent and alternative description framework of the Notched Stick, as previously discussed. In this case the horizontal axis of the Notched Stick has the role of opposing to the gravity force and the effect of the rotation produces, through the friction, a component force along the nail, eventually resulting in a horizontal displacement.

Qualitatively, static friction helps the onset of rotational motion because (1) it makes possible the momentum trasfer from the nail to the propeller and (2) it yields no energy dissipation, since there is no sliding of the propeller on the nail surface. In contrast, dynamic friction may oppose the onset of rotational motion, due to the dissipative nature of the force. So, it is conceivable that the best performances can be obtained by some intermediate values (neither too large nor too small) of static and dynamic dry friction coefficients. The experimental investigation of the role played by friction would have involved the use of different materials for the nail and the propeller hole, but such analysis was beyond the scope of the present work.

## Conclusions

Experimental and numerical results prove that the Notched Stick may act as a vibrot, a device able to convert an oscillatory motion (that of the stick) into a rotational one (that of the propeller). The effect turns out to be negligible, or does not occur at all, whenever the propeller is tightly connected to the stick nail and perfectly axisymmetrical with respect to the axis of the nail, so that the propeller center of mass lies on the symmetry axis of the nail. The small effects possibly observed can be probably attributed to friction forces. In contrast, the device succeeds in converting vibrations into rotations if the propeller center of mass is not aligned with the nail axis, a condition occurring when either the nail-propeller coupling is not tight or the propeller is not completely axisymmetrical relative to the nail axis. The propeller can be thought as a damped parametrically excited compound pendulum whose rotation may be induced by a process of parametric resonance for purely vertical oscillations of the nail, by ordinary resonance if the nail only oscillates horizontally or, finally, by a combination of both processes when nail oscillations take place in an intermediate direction (a conclusion that reasonably still holds in the more general case where a composition of two harmonic motions of the same frequency along orthogonal directions is imposed to the nail). Parametric resonance induces the onset of rotations also when the weight of the propeller can be regarded as negligible. The possible elliptical motion of the nail seems anyway unnecessary to determine the rotation of the propeller, since rotations are detected also in the case of a purely linear oscillation of the nail along any fixed direction. As an alternative description the device may be conceptualized as a horizontal axis hula-hoop (the propeller) moving around a horizontal body (the nail), but without any need of complex elliptic movements.

As a conclusion, although poorly efficient, the Notched Stick can be definitively regarded as a significant example of vibrot.
